# Exophthalmic Goitre

**Published:** 1890-03-22

**Authors:** 


### The Practitioner's Mirror and Retrospect.

*[The Editor will be glad to receive offers of co-operation and contributions from members of the profession. There is no doubt that much valuable material full of interest and instruction is at present lost to science. This is largely so in the case of* (1) *the younger and less-known members of the hospital staff* (2) *those connected with cottage hospitals, and* (3) *the great body of medical practitioners not attached to any institution. The co-operation of all is cordially invited to enable the Editor to make* The Hospital *of the utmost possible use and value to the profession. Specialists willing to review books on their special subjects are invited to communicate this fact at once. All letters should be addressed to* The Editor, The Lodge, Porchester Square, London, W.]

### MEDICINE.

### EXOPHTHALMIC GOITRE.

Exophthalmic goitre is one of the "studies of clinical medicine," contained in a recent number of Mr. Byrom Bramwell's periodical. There are four prominent symptoms of this malady, viz., increased frequency of the heart's action; enlargement of the thyroid gland; prominence of the eye-balls; and tremor. But in some cases there is no exophthalmos; in others there is no enlargement of the thyroid. Increased heart action is, however, always present, although at times more noticeable than at other periods. The subjects of this malady are usually eminently nervous, often affording a history of hereditary nervous tendency. But the subject of exophthalmic goitre has generally some affection of the digestive organs, such as tendency to vomiting, and sometimes jaundice; there is also often cough. Skin affections may occur, albumen or sugar may be found in the urine, and there is more or less well marked anæmia. It has also been noted that in cases of exophthalmic goitre there is diminished electrical resistance of the skin, a current passing through with greater facility than in health. This appears to be due to vasomotor alterations, in consequence of which the capillary vessels of the skin are dilated. In some cases profuse sweating is observed, and in others diarrhœa is a prominent symptom, occurring in irregular paroxysms. Pigmentation of the skin has also been observed, generally in those parts of the body where pigmentation is ordinarily most abundant. The tremor previously mentioned as a symptom, is a fine rhythmical vibratory tremor of the limbs, and sometimes of the body generally. In many cases the whole body can be felt to shake, if the hand is placed on the head or shoulder. Another symptom present in some cases was first described by the German oculist, Von Graefe. It consists, apparently, in a want of co-ordinate action between the eyeball and the upper lid. The upper lid fails to follow a downward movement of the eyeball, as it does in health. Dr. Bramwell thinks there can be no question that many subjects of exophthalmic goitre inherit a tendency to nerve disease. In many cases the parents have been affected with some form of nervous malady. The exciting cause seems in many cases to be fright, mental disturbance or strain, or grief and anxiety. Occasionally the exhaustion of acute illness seems to excite it. In a few cases there has been a history of head injury. Anything which lowers the nerve tone and produces exhaustion and debility may probably, in persons who are disposed, act as an exciting cause. Many of the persons affected with exophthalmic goitre are anæmic. Now, a prominent theory in the pathology of ophthalmic goitre is that which attributes it to morbid changes in the lower cervical ganglia of the sympathetic. Other views are, that the complaint is originated in some disturbance of the spinal cord, medulla oblongata, or brain. But, as a matter of fact, we really do not know what is the cause of exophthalmic goitre. It appears, however, that goitre, meaning enlargement of the thyroid gland, is a different disease from exophthalmic goitre. Dr. Bramwell appears to hold this view, and in Pepper and Starr's "Practice of Medicine," exophthalmic goitre is classed under the head of neurosis, and not as an affection of the thyroid gland, which, as Dr. Bramwell observes, is not always implicated. In countries—some parts of India, for instance—where goitre is endemic, exophthalmic goitre is not more common than in other parts of the country. The treatment of exophthalmic goitre is not very satisfactory. The condition of the system indicates tonics, and that of the heart indicates digitalis. Bromides are also useful. The eyes should be protected and shaded from glare. Good diet and freedom from mental strain must also be secured.

